# Meta-analysis of the prevalence of malaria associated with pregnancy in Colombia 2000–2020

**DOI:** 10.1371/journal.pone.0255028

**Published:** 2021-07-30

**Authors:** Jaiberth Antonio Cardona-Arias, Jaime Carmona-Fonseca

**Affiliations:** ”Grupo Salud y Comunidad César Uribe Pidrahíta”, University of Antioquia, Medellín, Colombia; Instituto Rene Rachou, BRAZIL

## Abstract

Knowledge about malaria associated with pregnancy is scarce in Latin America, and in Colombia, little is known about the magnitude of this infection. A systematic review was conducted to determine the prevalence of malaria associated with pregnancy (MAP) and each of its three forms: gestational (GM), placental (PM), and congenital (CM) tested using thick blood smear (TBS) and PCR. Also to compare the proportion of cases due *to Plasmodium falciparum* and *Plasmodium vivax* in Colombia from the year 2000–2020. We searched in Pubmed, Science Direct, EMBASE, EMCare, Cochrane Library, Scielo, Lilacs, Google Scholar, libraries, and repositories of Colombian universities, to obtain data on prevalence of GM, PM and CM with their respective testing method. We performed a meta-analysis with a random-effects model to obtain pooled prevalence of MAP and its three forms categorized by testing methods (TBS and PCR). We used data from 14 studies (out of 258 screened) contributing 7932, 2506 women for GM and PM respectively, also data on 1143 umbilical cord blood samples, and 899 peripheral blood of neonates. We found prevalence by TBS as, MAP 4.5% (95%CI = 2.9–6.9), GM 5.8% (95%CI = 3.8–8.7), PM 3.4% (95%CI = 1.7–6.7) and CM 1.3% (95%CI = 0.6–3.0). With PCR the prevalence was, MAP 14.4% (95%CI = 7.6–25.5), GM 16.7% (95%CI = 9.0–28.8), PM 11.0% (95%CI = 4.1–26.3) and CM 16.2% (95%CI = 8.2–29.5). The prevalence of submicroscopic infection was 8.5% (95%CI = 3.4–19.7) in GM, 10.1% (95%CI = 3.5–25.5) in PM and 22.0% (95%CI = 13.2–34.3) in CM. Infections by *P*. *vivax* was dominant over *P*. *falciparum* when tested with TBS, the PCR test gave similar proportions of *P*. *falciparum* and *P*. *vivax*. This meta-analysis has demonstrated high prevalence of MAP in Colombia, and highlights the urgent need to increase attention of researchers, research funding institutions, government agencies, and health authorities to study and intervene MAP, that has currently been under investigated.

## Introduction

The *World Malaria Report 2020* reported 229 million cases and 409,000 deaths, 94% in Africa [1]. There has been a notable increase in cases in America since 2015 due to outbreaks in Costa Rica, the Dominican Republic, and Ecuador and an increase in transmission in Brazil, Guyana, Nicaragua, Panama, and Colombia [2]. In Colombia, by epidemiological week 12 of 2021, 14,795 cases had been reported (in 2020, 65,293 were reported), 50.3% due to *Plasmodium vivax* (*P*. *vivax*), 49.0% due to *Plasmodium falciparum* (*P*. *falciparum*) and 0,7% due to mixed infections [3]. In the country, the incidence (adjusted annual parasite index/ 1,000 exposed) between 2000–2018 ranged between 3.90 and 11.50 (mean 6.52) with a stable trend, 821 municipalities with relatively good mosquito control, and 241 municipalities exposed [4]. Furthermore, between 2010–2014 there were 124,285 women with malaria, with 2.09% (2,596) reported as pregnant [5].

Malaria associated with pregnancy (MAP) includes the gestational malaria (GM), defined as the infection for *Plasmodium* spp. demonstrated in maternal peripheral blood by Thick Blood Smear (TBS), Polymerase Chain Reaction (PCR) or rapid diagnostic tests; the placental malaria (PM) which is defined with the presence of *Plasmodium* spp. in this organ by TBS, PCR, histopathology; and congenital malaria (CM) or infection in the neonate by transplacental transmission demonstrated in the first 7 days of life with a positive test for *Plasmodium* spp in umbilical cord or peripheral blood of the newborn. MAP is related to negative effects on the health of the pregnant woman (severe malaria, anemia, and death), fetus (anemia, intrauterine growth retardation, and death), and neonate; specifically in the pregnancy outcome can generate abortion, preterm labor, low birth weight and, death [6–8]. This highlights the importance of having robust epidemiological surveillance and investigative systems to diagnose, treat, monitor, and prevent new cases in pregnant women [8]. Despite its clinical, epidemiological, economic, and public health relevance in general, MAP is a relatively under-researched field, as shown in a systematic review that only found 617 original studies between 1925–2018, with a small number of studies in Latin America [9].

In Colombia, research on MAP is incipient, the few available studies have reported an increased risk of anemia, severe malaria with complications such as liver dysfunction, acidosis, and severe thrombocytopenia; lower birth weight and no record of neonatal or fetal deaths [10–12]. Furthermore, there are few studies that determine the magnitude of the problem. In this regard, previous investigations with TBS have reported prevalences of GM between 1.2% and 16.8% [13, 14]; of PM between 0.0% and 12.8% [14, 15]; of CM between 0.0% and 2.7% [12, 16]. While with PCR, these figures can amount to 32.1% in GM, 27.4% in PM [17], and 27.0% in CM [16], with differences in parasitic species involved, depending on the area of the country where the study is being carried out.

Heterogeneity in the magnitude of the MAP, the high variability of the available evidence with TBS or PCR, the low sample size used in some studies, the lack of knowledge of the national landscape on the prevalence of MAP, and the absence of meta-analysis in this field, demonstrate the need to conduct a systematic review on the prevalence of MAP in Colombia. The following arguments are added to the above: *i*) a systematic review allows to group the national evidence, improves the quality of the inferences and the accuracy of statistical estimates; *ii*) it is necessary to have a synthesis of the magnitude of the GM, PM, and CM, as an input for the orientation of subsequent etiological investigations and different prevention, care and surveillance actions of the disease; *iii*) meta-analyze the prevalence of submicroscopic MAP would generate solid evidence on the need to improve screening, diagnosis, and treatment programs; *iv*) this type of studies generates information on the global burden of the disease, its geographical distribution, variation between subgroups and changes in temporal trends; and *v*) generate faster evidence than a multicenter study when these are complex or too expensive [18].

The objectives of this research were: to analyze the general prevalence of MAP and specific of GM, PM, and CM according to TBS and PCR; estimate the prevalence of submicroscopic MAP, GM, PM, and CM; compare the proportion of cases due to *P*. *falciparum* and *P*. *vivax*; and identify some factors associated with MAP in Colombia 2000–2020, based on studies reported in the world scientific literature.

## Materials and methods

### PICO question: Population Intervention Comparison Outcome

Population: Pregnant women, parturients, and their placentas or neonates in whom tests for the diagnosis of MAP were applied. The subjects included in the different systematized studies met the following inclusion criteria: being a permanent resident in the endemic area for more than one year, pregnant women in good general health (with data from the clinical history, diseases or complications of the pregnant women were ruled out), absence of antimalarial treatment prior to study entry, hospital delivery, neonates diagnosed with GM (during pregnancy or in childbirth) and signature of the informed consent or assent by the mother.

Intervention: Per se, prevalence studies do not implement an intervention, but in this review, it would correspond to the application of TBS as a diagnosis of MAP.

Comparison: it would correspond to the implementation of PCR as a diagnostic method (although the first studies of the prevalence of MAP only applied TBS).

Outcome: Prevalence of MAP and its associated factors.

### Type of study

A systematic review of the literature and meta-analysis of prevalences [19, 20] (S1 Table).

#### Data source and searches

We searched in six multidisciplinary databases, including those that concentrate the most significant number of results in health sciences and biology: PubMed, EMCare, Science-Direct, Scielo, Lilacs, and Cochrane. Given the low publication on MAP in Colombia, the searches were complemented with queries in Google Scholar and in the institutional repositories of the main universities in Colombia with a track record in malaria research: Universidad de Antioquia, Universidad Nacional, Universidad De los Andes, Universidad De Córdoba, Universidad Del Valle, Universidad De Caldas and Universidad Del Pacífico. For the selection of the search terms, a pearl harvesting [21] was carried out in previous reviews and articles about MAP and was complemented with a query in the thesauri DeCS (in Spanish “*Descriptores en Ciencas de la Salud”*) and MESH *(Medical Subject Headings*). With these strategies, four terms were identified for pregnancy and three for malaria, and they were combined with the Boolean operator & for a total of 72 (12 syntaxes *6 databases) different search strategies, which were applied in each database (S2 Table). For the searches in the university repositories, since they did not contain good filters, only the term malaria, paludism (Plasmodium infections), or *Plasmodium* was searched, manually selecting the investigations on MAP.

The search had no restrictions on the year of publication, this review was delimited from the year 2000 because no publications were found prior to this decade (two studies from the last century were not available in full text). The identified studies were exported to a common source in the public reference manager *Zotero* to eliminate duplicates.

#### Eligibility criteria

Three inclusion criteria were applied:

1. It had to be an original study, thus eliminating reviews, editorials, and book chapters
2. Be a study on MAP, reporting pregnant women, parturient, and their placentas or neonates in whom tests for the diagnosis of MAP were applied.
3. Studies that reported the prevalence of infection

It is worth clarifying that the term "prevalence" was not included in the search syntax since doing so resulted in a lower search sensitivity (very few studies were found). Therefore, it was decided to do a broader search (excluding this term from the syntax) and then select the prevalence studies using the last inclusion criterion.

The exclusion criteria were studies that were not carried out in Colombia or multicenter studies that included a group of pregnant women from Colombia, but without presenting the specific data for this country; studies that took data from previously published studies without including new information; and articles not available on the web and for which no response was obtained from the authors via email.

#### Study selection and data extraction

The included studies were systematized using a qualitative synthesis of the variables title, authors, year and location of the study, type of MAP studied (gestational, placental, or congenital), number of study subjects, number of positives per diagnostic test (TBS or PCR) and species (*P*. *falciparum*, *P*. *vivax*, and mixed infections) and associated factors.

A MAP expert validated the protocol, the reproducibility was evaluated during the selection of the studies, and the extraction of the information by two investigators (JACA and JCF) who settled any differences by consensus.

### Quality assessment

The methodological quality was assessed using the STROBE guideline (*Strengthening the Reporting of Observational Studies in Epidemiology*) (S3 Table); which contains 3 items to evaluate the quality of the title-abstract, background-rationale, and objectives; 8 criteria to determine methodological quality (study design, setting, selection of participants, variables, data source, bias, study size and statistical methods), 5 items for the results, and 5 for the discussion, limitations, interpretation, generalizability and funding.

### Data analysis

The variables were described with frequencies. The following prevalences of infection were determined with a 95% confidence interval:

1. Prevalences of MAP as the sum of all positive over all tested samples, expressed in percentage, independent of the compartment analyzed (peripheral blood of the pregnant woman, the placenta, the cord, or the neonate)
2. Prevalences of GM, PM, and CM
3. Prevalences according to Thick Blood Smear and CRP
4. Based on the studies that applied both diagnostic tests, the prevalence of submicroscopic infection (positive with PCR and negative with TBS) was determined
5. Frequencies by parasitic species

A random effect model was applied to obtain pooled prevalence of MAP in its three forms (GM, PM and CM) categorised per testing method (TBS and PCR). Heterogeneity was evaluated using Q, I-squared, and Tau-squared tests; publication bias was evaluated using Begg’s statistical test, and Funnel plot; sensitivity analysis was evaluated using influence diagrams to determine the effect of each study on the pooled measure, and Forest Plot for reporting the combined and individual prevalence of each study.

The analyzes were carried out in Epidat 3.1, Excel, and SPSS 25.0, taking p values less than 0.05 as significant.

## Results

**Fig 1:**
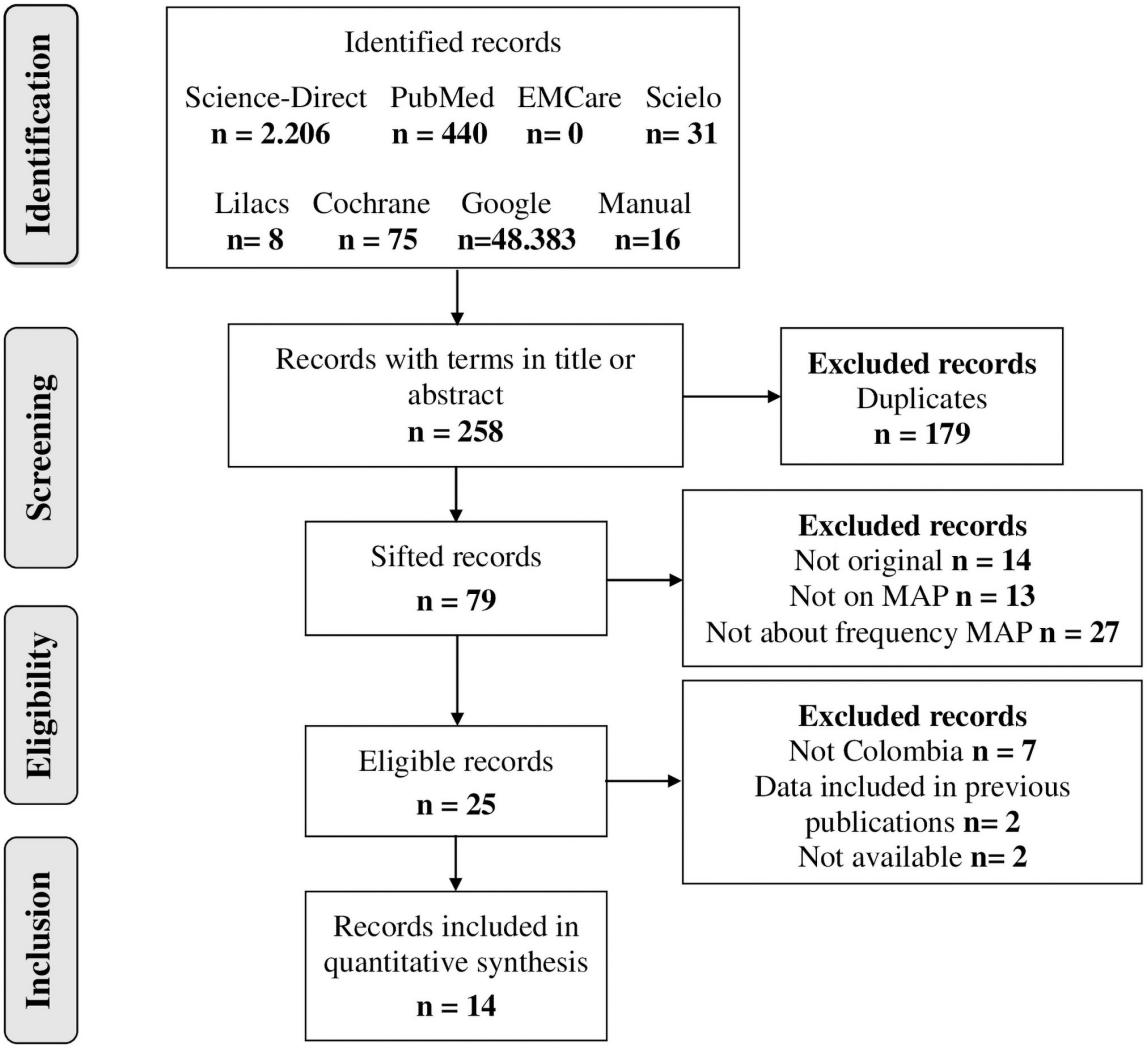
Flowchart of the search and selection protocol of studies about the prevalence MAP in Colombia.

In the initial search, without applying any filters in the databases, 51,159 results wereidentified, of which only 79 were screened after eliminating duplicates. After the eligibility criteria were applied, only 14 investigations were obtained that complied with the protocol (Fig 1).

**Fig 2:**
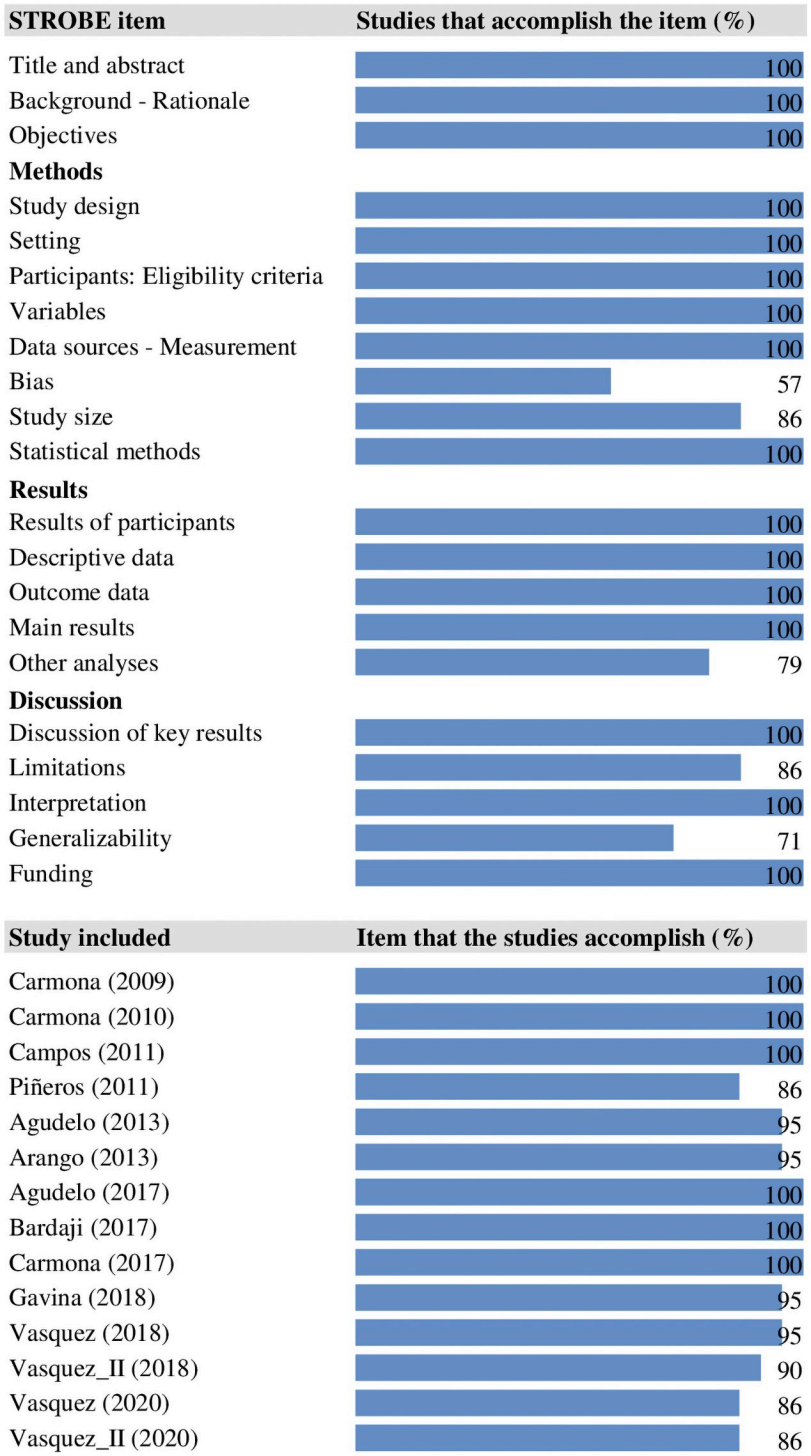
Assessment of the methodological quality of the included studies.

All the included studies showed excellent methodological quality, meeting more than 80% of the criteria of the STROBE guideline. However, only 57% explicitly explained how the bias control was carried out (in those that did not explain it, due to the type of diagnostic tests and eligibility criteria applied, it was inferred that the risk of selection and information bias was low) (Fig 2).

The studies were published between 2009 and 2020 (the two unavailable studies corresponded to a report on MC from 1985 and one on GM in hospitalized patients from 1996), the majority were carried out in rural areas of the northwest of the country (except for the studies by the Vásquez’s group that included pregnant women from urban areas of the Colombian Pacific region). 93% of studies (n = 13) determined the prevalence of GM with 7932 women, 86% (n = 12) of PM with 2506 placentas, and 50% (n = 7) of CM with 1143 umbilical cord samples and with 899 peripheral blood from neonates (Table 1).

**Table 1:** Description of the included studies according to the year of publication, location of the study, type of MAP, and the number of subjects.

Author	Year	Location	Type of MAP	N
Carmona [12]	2009	Turbo, Necoclí, Carepa	GM PM CM	2450
Carmona [14]	2010	Turbo, Necoclí, Carepa	GM PM CM	386
Campos [17]	2011	Turbo, Necoclí, Carepa	GM PM CM	252
Piñeros [22]^a^	2011	Apartadó, Carepa, Turbo, Necocli	PM CM	290
Agudelo [23]	2013	Turbo, Puerto Libertador	GM PM	242
Arango [24]	2013	Turbo, Necoclí, Puerto Libertador	GM PM CM	288
Agudelo [16]	2017	Tierralta, Puerto Libertador	GM PM CM	411
Bardaji [13]	2017	Tierralta	GM PM CM	3914
Carmona [25]	2017	Turbo, Necoclí, Puerto Libertador	GM	91
Gavina [15]	2018	Puerto Libertador	GM PM	369
Vásquez [26]	2018	El Bagre, Quibdó, Tumaco	GM PM	787
Vásquez II [27]	2018	El Bagre, Quibdó, Tumaco, Apartadó	GM PM	1026
Vásquez [10]	2020	Apartadó,Turbo, El Bagre, Quibdó, Tumaco	GM PM	1116
Vásquez II [28]	2020	Quibdó, Tumaco	GM	858

^a^ This research took part of the data previously published by Carmona-2009, it has the same funder. It took the data from the three municipalities of the Carmona study, adds one more municipality, but since it did not disaggregate the data by municipality, it was not possible to exclude from the analyzes part of the data that was in duplicated.

### Prevalence of MAP

With TBS, prevalences of MAP was found to be between 0.7% and 11.1% A random-effects model showed a pooled prevalence of 4.5% (95%CI = 2.9–6.9). Similarly, for PCR, the pooled prevalence was 14.4% (95%CI = 7.6–25.5) (Table 2). In the sensitivity analysis for the TBS studies, none had greater weight on the combined measure. However, in the measurements with PCR, differences were found when eliminating each study in successive phases. When the Vásquez studies were excluded from the analysis, the prevalence of MAP rose to 28.1% (95%CI = 26.1–30.3), which could be explained by the type of PCR used in their studies.

**Table 2:** Prevalence of malaria associated with pregnancy reported in individual studies and combined measure.

Author	Thick blood smear (TBS)		Polymerase chain reaction (PCR)	
	Event (%)	95% CI	Event (%)	95% CI
Carmona [12]	9,9	8,8–11,2	ND	No data
Carmona [14]	11,1	8,4–14,7	ND	No data
Campos [17]	8,3	5,5–12,4	24,2^a^	19,3–29,9
Piñeros [22]	7,6	5,0–11,3	ND	No data
Agudelo [23]	6,2	3,8–10,0	15,3 ^a^	11,3–20,4
Arango [24]	7,0	4,6–10,6	45,1 ^b^	39,5–50,9
Agudelo [16]	3,6	2,2–6,0	39,2 ^b^	34,6–44,0
Bardaji [13]	0,7	0,5–10,0	6,6 ^b^	4,7–9,1
Carmona [25]	7,7	3,7–15,3	45,1 ^b^	35,2–55,3
Gavina [15]	1,9	0,9–2,9	17,9 ^b^	14,3–22,1
Vásquez [26]	4,2	3,0–5,8	6,1 ^a^	4,6–8,0
Vásquez II [27]	2,8	2,0–4,0	4,2 ^a^	3,1–5,6
Vásquez [10]	3,1	2,3–4,3	4,6 ^a^	3,5–6,0
Vásquez II [28]	2,7	1,8–4,0	5,5 ^a^	4,1–7,2
**Meta-analysis**				
Fixed model	6,1	5,6–6,6	16,7	15,5–17,9
Random model	**4,5**	**2,9–6,9**	**14,4**	**7,6–25,5**
Q-value	296**		685**	
I-squared	96		98	
Tau-squared	0,70		1,42	

**<0,001.

^a^ Nested PCR.

^b^ Real-time quantitative PCR.

Using TBS, the prevalence of GM was 5.8% (95%CI = 3.8–8.7), of PM 3.4% (95%CI = 1.7–6.7), and of CM 1.3% (95%CI = 0.6–3.0), without finding a greater weight of one of the studies according to the sensitivity analysis. Meanwhile, the pooled prevalences using PCR were GM 16.7% (95%CI = 9.0–28.8), PM 11.0 (95%CI = 4.1–26.3) and CM 16,2% (95%CI = 8.2–29.5) (Table 3), with this diagnostic test, the sensitivity analysis showed changes when eliminating some studies; Therefore, by excluding the Vásquez studies from the pooled measure, the prevalence of GM rose to 30.0% (95%CI = 27.0–33.2) and of PM to 33.5% (95%CI = 29.3–37.9). In CM, when excluding the Bardaji study (this study processed samples of women with and without GM, while the others only included infants with diagnoses of GM), the prevalence was 2.2% (95%CI = 1.3–3.6) with TBS and 23.1% (95%CI = 15.2–33.5) with PCR.

**Table 3:** Prevalence of gestational, placental, and congenital malaria reported in individual studies and the combined measure, according to the diagnostic test.

Author	TBS^a^ - Event % (95% CI)			PCR^b^ - Event % (95% CI)		
	GM^c^	PM^d^	CM^e^	GM^e^	PM^d^	CM^e^
Carmona [12]	10,4 (9,2–11,8)	12,0 (7,7–182)	2,7 (1,1–6,4)	No data	No data	No data
Carmona [14]	16,8 (11,5–23,8)	12,8 (8,1–19,6)	1,8 (0,5–7,0)	No data	No data	No data
Campos [17]	13,1 (7,4–22,1)	9,5 (4,8–17,9)	2,4 (0,6–9,0)	32,1 (23,1–42,8)	27,4 (18,9–37,9)	13,1 (7,4–22,1)
Piñeros [22]	9,1 (5,1–15,7)	18,1 (11,5–27,2)	2,6 (1,1–6,0)	No data	No data	No data
Agudelo [23]	12,6 (7,3–20,9)	3,3 (1,2–8,5)	No data	14,0 (8,9–21,4)	16,5 (10,9–24,2)	No data
Arango [24]	8,0 (4,5–13,9)	8,4 (4,3–15,9)	0,5 (0,0–7,8)	49,0 (39,1–58,9)	57,3 (47,2–66,8)	29,2 (21,0–39,0)
Agudelo [16]	1,2 (0,8–1,7)	2,9 (1,1–7,5)	0,4 (0,0–5,5)	39,4 (31,6–47,8)	51,1 (42,8–59,4)	27,0 (20,2–35,0)
Bardaji [13]	7,7 (3,7–15,3)	0,5 (0,2–1,5)	0,1 (0,0–0,6)	9,0 (6,2–12,8)	3,0 (1,0–8,9)	3,0 (1,0–8,9)
Carmona [25]	3,7 (1,8–7,6)	No data	No data	45,1 (35,2–55,3)	No data	No data
Gavina [15]	5,8 (4,1–8,2)	0,3 (0,0–4,2)	No data	30,5 (24,3–37,4)	4,9 (2,6–9,2)	No data
Vásquez [26]	3,7 (2,5–5,3)	0,8 (0,0–3,1)	No data	7,3 (5,4–9,9)	3,5 (1,8–6,6)	No data
Vásquez II [27]	4,2 (3,0–5,8)	0,7 (0,0–2,7)	No data	4,7 (3,4–6,5)	2,8 (1,4–5,4)	No data
Vásquez [10]	2,7 (1,8–4,0)	0,6 (0,0–2,4)	No data	5,3 (4,0–7,1)	2,7 (1,4–5,2)	No data
Vásquez II [28]	2,7 (1,8–4,0)	No data	No data	5,5 (4,1–7,2)	No data	No data
**Meta-analysis**						
Fixed	6,8 (6,3–7,5)*	7,7 (6,2–9,4)*	1,8 (1,1–2,9)*	14,6 (13,4–16,0)*	22,5 (19,6–25,7)*	22,7 (18,5–27,5)*
Random	5,8 (3,8–8,7)*	3,4 (1,7–6,7)*	1,3 (0,6–3,0)*	16,7 (9,0–28,8)*	11,0 (4,1–26,3)*	16,2 (8,2–29,5)*
Q-I-Tau	217-94-0,63	96-89-1,3	14-55-0,62	419-98-1,4	282-97-2,5	23-87-0,5

*<0,001.

^a^TBS: Thick blood smear.

^b^PCR: Polymerase chain reaction.

^c^Gestational malaria.

^d^Placental malaria.

^e^Congenital malaria.

The prevalence of submicroscopic infection was 8.5% (95%CI = 3.4–19.7) in GM, 10.1% (95%CI = 3.5–25.5) in PM and 22.0% (95%CI = 13.2–34.3) in CM (Table 4). The prevalence of submicroscopic MAP was 9.1% (95%CI = 4.0–19.3); By excluding the Vásquez studies, the submicroscopic infection of MAP amounted to 23.3% (95%CI = 14.7–34.8), that of GM to 24.7% (95%CI = 16.9–34,5) and that of PM to 22.0% (95%CI = 9.1–44.1).

**Table 4:** Meta-analysis of the prevalence of submicroscopic malaria associated with pregnancy.

Author	GM^a^	PM^b^	CM^c^
	Event % (95% CI)		
Campos [17]	19,0 (12,0–28,9)	17,9 (11,1–27,5)	10,7 (5,-19,3)
Agudelo [23]	5,0 (2,2–10,6)	13,2 (8,3–20,5)	No data
Arango [24]	36,8 (27,8–47,0)	49,5 (39,6–59,4)	29,5 (21,2–39,4)
Agudelo [16]	31,4 (24,2–39,6)	48,2 (39,9–56,5)	27,0 (20,2–35,0)
Carmona [25]	37,4 (28,1–47,7)	No data	No data
Gavina [15]	26,7 (20,9–33,5)	4,9 (2,6–9,2)	No data
Vásquez [26]	1,5 (0,8–3,0)	2,7 (1,3–5,6)	No data
Vásquez II [27]	1,1 (0,5–2,2)	2,1 (0,9–4,5)	No data
Vásquez [10]	1,1 (0,6–2,2)	2,1 (1,0–4,4)	No data
Vásquez II [28]	2,8 (1,9–4,1)	No data	No data
**Meta-analysis**			
Fixed	14,7 (13,0–16,7)**	21,3 (18,4–24,6)**	24,7 (20,1–29,9)**
Random	8,5 (3,4–19,7)**	10,1 (3,5–25,5)**	22,0 (13,2–34,3)**
Q-I-Tau	370-98-2,4	236-97-2,5	10-79-0,3

**<0,01.

^a^Gestational malaria.

^b^Placental malaria.

^c^Congenital malaria.

### Cases per malaria species

Excluding the studies by Vásquez (because in the sensitivity analyzes they modified the pooled measure), the distribution by species was as follows:

1. With TBS: *i*) MAP by *P*. *vivax* 75,4% (306/406), *P*. *falciparum* 23,4% (95/406) and by mixed infections 1,2% (5/406); *ii*) GM by *P*. *vivax* 75,3% (238/316), *P*. *falciparum* 23,1% (73/316) and by mixed infections 1,6% (5/316); *iii*) PM by *P*. *vivax* 74.7% (56/75) and *P*. *falciparum* 25,3% (19/75); and iv) CM by *P*. *vivax* 85.7% (12/14) and *P*. *falciparum* 14,3% (2/14).
2. With PCR: *i*) MAP by *P*. *vivax* 45,9% (243/529), *P*. *falciparum* 47.8% (253/529) and by mixed infections 6.2% (33/529); *ii*) GM by *P*. *vivax* 53,0% (143/270), *P*. *falciparum* 43.0% (116/270) and by mixed infections 4.0% (11/270); *iii*) PM by *P*. *vivax* 40,0% (72/180), *P*. *falciparum* 49.4% (89/180) and by mixed infections 10.6% (19/180); and iv) CM by *P*. *vivax* 36,8% (28/76), *P*. *falciparum* 59.2% (45/76) and by mixed infections 3.9% (3/76).

**Fig 3:**
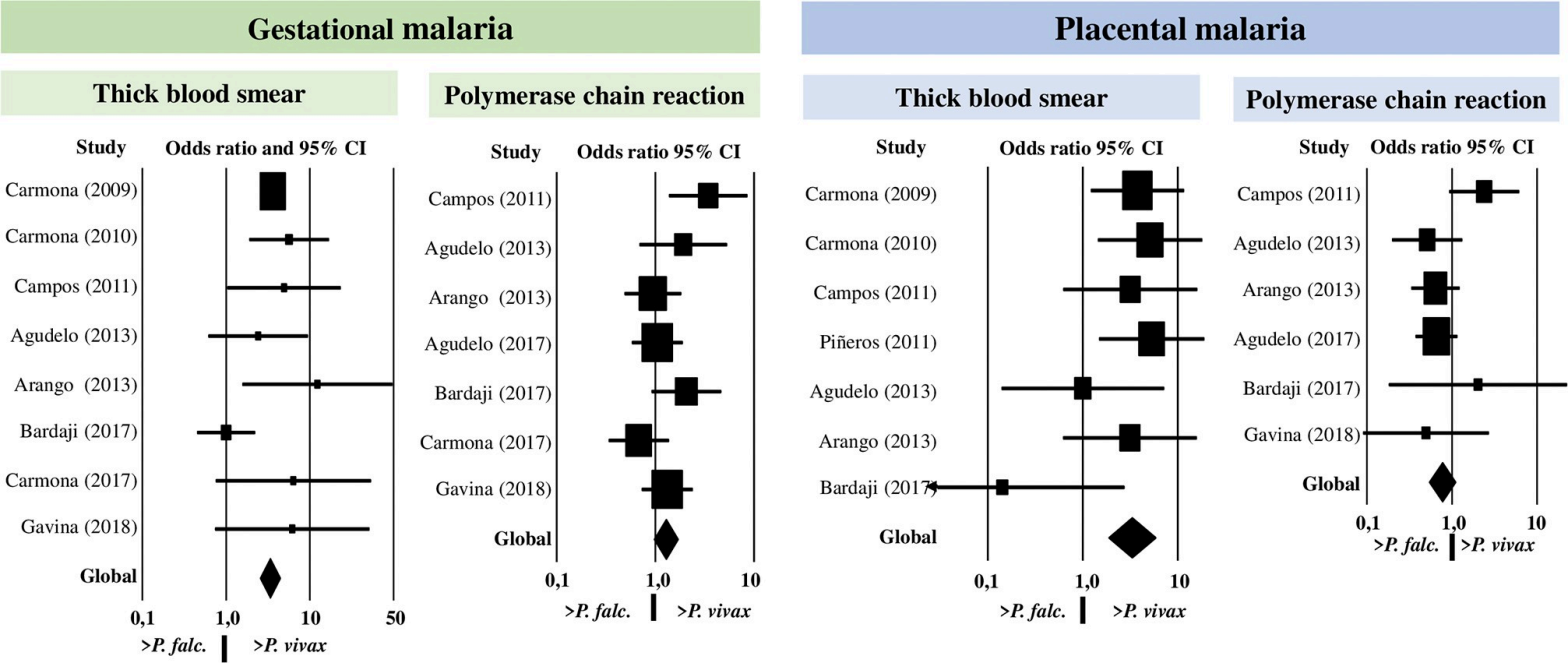
Meta-analysis for the comparison of the prevalence of *Plasmodium vivax* vs. *Plasmodium falciparum*.

**Fig 4:**
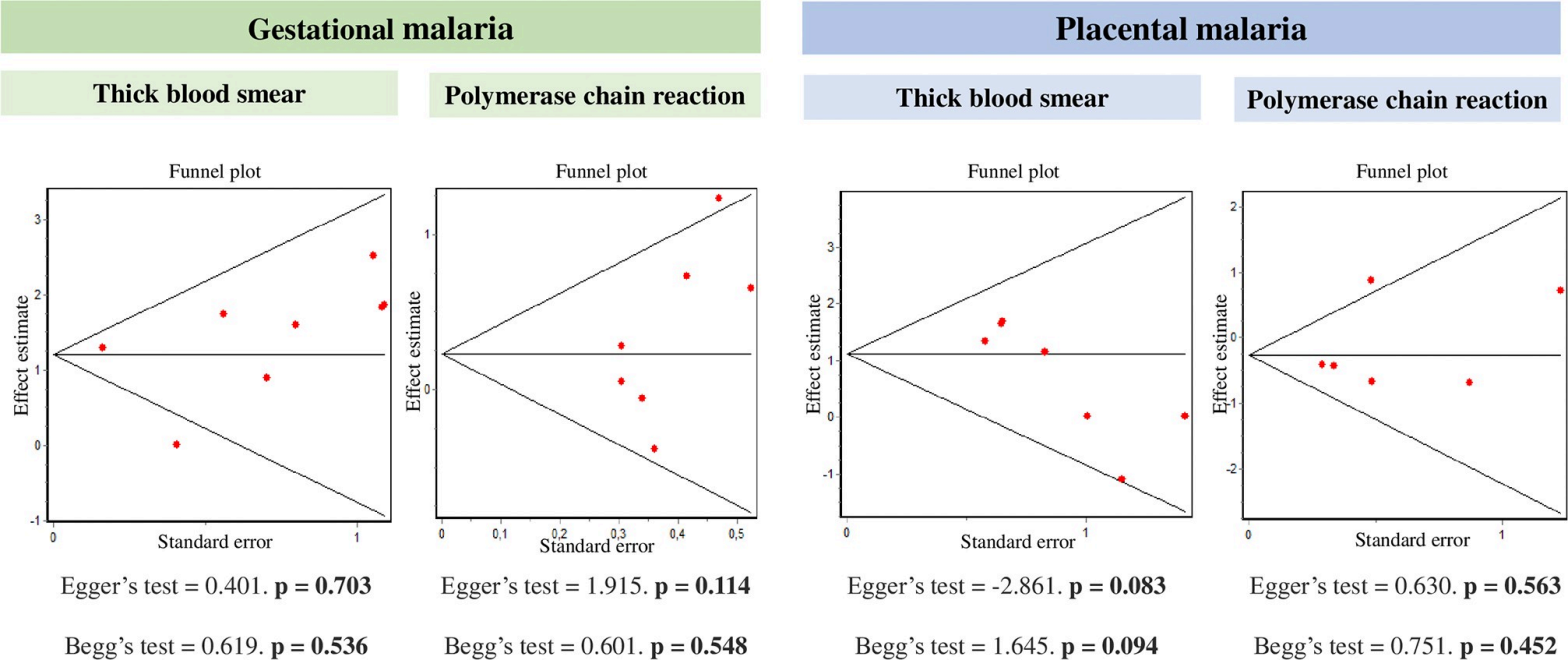
Assessment of publication bias.

When comparing the prevalence of MAP caused by *P*. *vivax* vs. *P*. *falciparum*, excluding the studies by Vásquez, a higher proportion of *P*. *vivax* cases was found with TBS, in GM with an OR of 3.3 (95%CI = 2.5–4.4) and in PM of 3.3 (95%CI = 1.8–5.7), without finding any differences in the distribution of these two species with PCR since for GM the OR was 1.3 (95% CI = 0.9–1.7), and in PM 0.8 (0.5–1.1) (Fig 3). In these meta-analyzes, no publication bias was found, as observed with the graphical method and the Begg and Egger statistics (Fig 4). With TBS, the comparison of both species presented the following results: MAP registered an OR of 3.3 (95%CI = 2.6–4.2) with *P*. *vivax* being higher, CM did not show differences in the prevalence of both species with an OR 3.1 (95%CI = 0,9–10.1). With PCR, the comparison of both species presented the following results: MAP registered an OR of 1.0 (95%CI = 0.7–1.5) and CM 0.7 (95%CI = 0.5–1.1) that indicated a similar prevalence of both species.

Finally, among the factors associated with MAP, the area of residence (higher GM in rural areas) [10]; the number of previous pregnancies (more GM in women with a higher number of pregnancies) [12]; the weeks of gestation (lower gestational age in infected women) [10, 12]; the number of malaria episodes in the current pregnancy [10, 23], in the last year and among partners (higher among infected pregnant women) [10]; and lower birth weight were reported [12]. However, this evidence was not consistent among the studies (even between those carried out in the same endemic area), since other authors did not report statistical differences in MAP according to maternal age [10, 15, 16, 22, 24], gestational age [15, 16, 24], number of pregnancies [15, 22], previous episodes of malaria [16, 22], parity [16, 23, 24], weight and height of the newborn [15, 16, 22, 24], neonatal anemia, or preterm delivery [15].

## Discussion

We carried out a metanalysis to determine prevalence of MAP in Colombia using data from 7932 pregnant women, 2506 placenta samples, 1143 umbilical cord samples, and 899 peripheral blood samples from neonates. The selected studies were carried out from nine municipalities with high endemicity for malaria in Colombia. This constituted a study with greater possibilities of generalization of results and better statistical precision of the estimates of the different prevalences of MAP exposed, thereby materializing some of the main advantages of this type of research [18].

Using TBS, the prevalence of GM was 5.8% (95%CI = 3.8–8.7), of PM 3.4% (95%CI = 1.7–6.7) and of CM 1.3% (95%CI = 0.6–3.0), which increase with the use of PCR to 16.7% (95%CI = 9.0–28.8), 11.0% (95%CI = 4.1–26.3) and 16.2% (95%CI = 8.2–29.5) respectively, showing a higher diagnostic yield of PCR to detect cases. This outcome is different from the findings of a meta-analysis carried out with 49 studies, which indicated that PCR and light microscopy generate similar diagnostic results. However, this same study declares as a limitation the fact that the highest proportion of systematized studies were carried out in infections by *P*. *falciparum*, for which more investigations are required to determine the diagnostic yield of these tests for areas with a predominance of *P vivax*. Likewise, in the case of PM diagnosis, it is recommended to standardize placental histology as a reference diagnostic test and to improve studies on the possible false-positives of PCR (when histology is taken as standard) or to determine if the positive results of PCR and negative in histology are explained by the presence of sequestered parasites in the placenta that are not detected by microscopy [29].

Regardless of the limitations in the diagnostic yield or the operational complexity inherent to the implementation of TBS and PCR, this meta-analysis showed a high prevalence of GM, PM, and CM, demonstrating a high risk of parasite transmission with the subsequent risk of maternal anemia, cerebral malaria, abortion, stillbirth, preterm delivery, low birth weight, infant mortality and anemia [7], among other outcomes with serious medical, epidemiological, economic and public health impacts, which supports the need to increase the resources allocated to the early diagnosis and timely treatment of MAP.

The high prevalence of MAP reported in this research is more serious when considering the low amount of resources allocated to malaria control in Colombia. An amount corresponding to less than US$10 million for diagnosis, treatment, surveillance, promotion, and prevention activities as part of the framework of the Regional Initiative for the Elimination of Malaria (*Iniciativa Regional para la Eliminación de la Malaria*) “*IREM*,” with no specific lines of action specified for pregnant women and their children [30].

The prevalence of submicroscopic infection was 8.5% (95%CI = 3.4–19.7) for GM, 10.1% (95%CI = 3.5–25.5) for PM, and 22.0% (95%CI = 13.2–34.3) for MC, similar figures to those found in a systematic review of submicroscopic infections with studies from Africa, where it was 36% [31]. These results are worrying considering that the clinical practice guidelines for diagnosing and treating malaria in Colombia only consider TBS and some rapid tests, which means that these submicroscopic infections would not be diagnosed or treated [32]. In addition, undiagnosed submicroscopic infections, as well as asymptomatic ones, constitute an obstacle to the control and elimination programs since they allow the permanence of reservoirs of the parasite and thereby determining the intensity and stability of malaria transmission, especially in regions with low endemicity, as documented in previous systematic reviews [33]. Consequently, all of the above highlights the urgency of implementing more sensitive diagnostic tests and increasing financial resources to detect and treat this type of infection.

Using TBS, *P*. *vivax* infections predominated, and with PCR, the proportions of *P*. *falciparum* and *P*. *vivax* were statistically similar, which differs from the majority of previous studies on MAP in which *P*. *falciparum* predominates [9, 29, 31]. These findings evidence the need to improve the funding of studies in Northwest Colombia, where *P*. *vivax* infections represent a high proportion, allowing researchers to investigate the specificity of epidemiological and pathological factors and the clinical effects of MAP caused by this species. Additionally, a previous systematic review with 59 studies estimated that GM *P*. *vivax* increased the odds of fetal death by 2.8 when detected at delivery; these odds are reduced to 1.09 when the infection was detected and treated during pregnancy [34].

Finally, it is essential to note that the report of the factors associated with MAP was deficient. Some studies reported the association of MAP with the number of pregnancies [14], gestational age [10, 12], previous malaria episodes [10, 23], and birth weight [12]. However, these findings were not consistent between the studies, so their research should be circumscribed to more local spaces, which allow the identification of possible risk factors specific to each group. Likewise, it is important to specify that the greatest proportion of studies focused on parasitological variables, which allows us to suggest that in Colombia a further development of epidemiological studies is required to improve the identification of risk factors, increase the number of sociodemographic, clinical and epidemiological variables analyzed, and develop subsequent etiological studies to quantify the risk factors of GM, PM, and CM.

Consistent with the above, the limitations of this research include not being able to perform meta-regressions or subgroup analyzes, which allow optimizing malaria elimination strategies, such as the comparison of pooled prevalence with general population, non-pregnant women, by gravidity, and other associated factors. Despite these limitations, the findings of this meta-analysis allow to suggest the following strategies for programmatic implementation of MAP elimination in the affected communities: use molecular diagnostic tests to detect submicroscopic cases, implement active malaria surveillance systems at the beginning of pregnancy to reduce the prevalence of PM and MC, increase the budget for primary prevention actions in pregnant women and their families, create a specific budget line for the prevention and control of MAP in malaria and prenatal control programs.

## Conclusion

This meta-analysis demonstrated a high prevalence of MAP and its three forms GM, PM and GM; underestimation of prevalence with TBS; very high prevalence of submicroscopic infections; similarity in the proportion of *P*. *falciparum* and *P*. *vivax*; and little research on the factors associated with MAP and its effects on maternal, fetal and infant health. It is necessary to develop studies for risk factors for MAP in order to have proper ways to understand and control this health problem. These results, together with the absence of specific objectives in Colombia’s National Malaria Strategic Plan 2019–2022, highlight the urgent need to increase the efforts of researchers, research funding institutions, and government agencies and health authorities (WHO, PAHO, UNICEF, and others) to study MAP and its forms, in order to advance its knowledge and subsequent control.

## Supporting information

S1 Table PRISMA checklist. (DOCX)

S2 Table Search strategies applied. (DOCX)

S3 Table Methodological quality (risk of bias) assessment. (XLSX)

## Abbreviations

CM: Congenital Malaria
DeCS: Descriptores en Ciencias de la Salud
GM: Gestational Malaria
MAP: Malaria Associated with Pregnancy
MESH: Medical Subject Headings
P. (*falciparum* or *vivax*): *Plasmoduim*
PCR: Polymerase Chain Reaction
PICO: Population Intervention Comparator Outcome
PM: Placental Malaria
PRISMA: Preferred Reporting Ítems for Systematic Reviews and Meta-Analyses
STROBE: Strengthening the Reporting of Observational Studies in Epidemiology
TBS: Thick Blood Smear
