# Supplementary material for: Meta-analysis of the prevalence of malaria associated with pregnancy in Colombia 2000–2020
Source: PLoS One. 2021 Jul 30;16(7):e0255028. doi: 10.1371/journal.pone.0255028 (PMC8323879; doi:10.1371/journal.pone.0255028)
Supplement: S2 Table — (DOCX) [file pone.0255028.s002.docx]

**Search strategies applied.**

| PubMed^a^ |
| --- |
| 1. (malaria & gestation[Title/Abstract]) AND (Colombia) |
| 1. (malaria & embarazo[Title/Abstract]) AND (Colombia) |
| 1. (malaria & pregnancy[Title/Abstract]) AND (Colombia) |
| 1. (malaria & placenta[Title/Abstract]) AND (Colombia) |
| 1. (paludism & gestation[Title/Abstract]) AND (Colombia) |
| 1. (paludism & embarazo[Title/Abstract]) AND (Colombia) |
| 1. (paludism & pregnancy[Title/Abstract]) AND (Colombia) |
| 1. (paludism & placenta[Title/Abstract]) AND (Colombia) |
| 1. (plasmodium & gestation[Title/Abstract]) AND (Colombia) |
| 1. (plasmodium & pregnancy[Title/Abstract]) AND (Colombia) |
| 1. (plasmodium & embarazo[Title/Abstract]) AND (Colombia) |
| 1. (plasmodium & placenta[Title/Abstract]) AND (Colombia) |
| Science Direct^b^ |
| 1. Find articles with these terms: Colombia. Title, abstract, keywords: Malaria & gestation |
| 1. Find articles with these terms: Colombia. Title, abstract, keywords: Malaria & embarazo |
| 1. Find articles with these terms: Colombia. Title, abstract, keywords: Malaria & pregnancy |
| 1. Find articles with these terms: Colombia. Title, abstract, keywords: Malaria & placenta |
| Cochrane^b^ |
| 1. Trial matching Colombia Malaria Gestation in Title Abstract Keyword |
| 1. Cochrane Reviews matching Colombia Malaria embarazo in Title Abstract Keyword |
| 1. Trial matching Colombia Malaria pregnancy in Title Abstract Keyword |
| 1. Cochrane Reviews matching Colombia Malaria placenta in Title Abstract Keyword |
| Scielo^b^ |
| 1. (ab:(Colombia & Malaria & gestation)) |
| 1. (ab:(Colombia & Malaria & embarazo)) |
| 1. (ab:(Colombia & Malaria & pregnancy)) |
| 1. (ab:(Colombia & Malaria & placenta)) |
| Lilacs^b^ |
| 1. Título, resumen, Asunto: Colombia & Malaria & gestation |
| 1. Título, resumen, Asunto: Colombia & Malaria & embarazo |
| 1. Título, resumen, Asunto: Colombia & Malaria & pregnancy |
| 1. Título, resumen, Asunto: Colombia & Malaria & placenta |
| Google Scholar^b^ |
| 1. allintitle: Colombia & Malaria & Gestation |
| 1. allintitle: Colombia & Malaria & embarazo |
| 1. allintitle: Colombia & Malaria & pregnancy |
| 1. allintitle: Colombia & Malaria & placenta |

The last search in all information sources was carried out on February 28, 2021.

^a^ The boolean operator OR was not applied for "malaria OR paludism OR Plasmodium" or for "gestation OR embarazo OR pregnancy OR placenta" since applying this syntax resulted in a much smaller number of results. Therefore it was decided to separate the terms into 12 searches with which the sensitivity was increased. ^b^ In order not to lengthen the table, the four search syntaxes using the term "paludism" are not included, nor the additional four with the term "Plasmodium.”
